# The effect of cognitive-based training for the healthy older people: A meta-analysis of randomized controlled trials

**DOI:** 10.1371/journal.pone.0176742

**Published:** 2017-05-01

**Authors:** Huei-Ling Chiu, Hsin Chu, Jui-Chen Tsai, Doresses Liu, Ying-Ren Chen, Hui-Ling Yang, Kuei-Ru Chou

**Affiliations:** 1 School of Nursing, College of Nursing, Taipei Medical University, Taipei, Taiwan; 2 Gangshan Branch of Armed Forces Kaohsiung General Hospital, Kaohsiung, Taiwan; 3 Aviation Physiology Research Laboratory, Kaohsiung Armed Forces General Hospital Gangshan Branch, Kaohsiung, Taiwan; 4 Institute of Aerospace and Undersea Medicine, School of Medicine, National Defense Medical Center, Taipei, Taiwan; 5 Department of Neurology, Tri-Service General Hospital, National Defense Medical Center, Taipei, Taiwan; 6 Department of Nursing, Taipei Medical University-Shuang Ho Hospital, Taipei, Taiwan; 7 Department of Nursing, Wan Fang Hospital, Taipei Medical University, Taipei, Taiwan; 8 Department of Nursing, Taoyuan Armed Forces General Hospital, Taoyuan, Taiwan; 9 Psychiatric Research Center, Taipei Medical University Hospital, Taipei, Taiwan; Nanjing Normal University, CHINA

## Abstract

**Background:**

From the perspective of disease prevention, the enhancement of cognitive function among the healthy older people has become an important issue in many countries lately. This study aim to investigate the effect of cognitive-based training on the overall cognitive function, memory, attention, executive function, and visual-spatial ability of the healthy older people.

**Methods:**

Cochrane, PubMed, EMBASE, MEDLINE, PsycINFO, and CINAHL of selected randomized controlled trials (RCTs), and previous systematic reviews were searched for eligible studies. The population focused on this study were healthy older people who participated in randomized controlled trials that investigated the effectiveness of cognitive-based training. The outcomes including change in overall cognitive function, memory, attention, executive function, and visual-spatial ability.

**Results:**

We collected a total of 31 RCTs, the results showed that cognitive-based training has a moderate effect on overall cognitive function (g = 0.419; 95%CI = 0.205–0.634) and executive function (g = 0.420; 95%CI = 0.239–0.602), and a small effect on the memory (g = 0.354; 95%CI = 0.244–0.465), attention (g = 0.218; 95%CI = 0.125–0.311), and visual-spatial ability (g = 0.183;95%CI = 0.015–0.352) in healthy older people. Subgroup analysis indicated the intervention characteristics of ≧3 times each week (p = 0.042), ≧8 total training weeks (p = 0.003) and ≧24 total training sessions (p = 0.040) yields a greater effect size.

**Conclusions:**

Cognitive-based training is effective for the healthy older people. This improvement can represent a clinically important benefit, provide information about the use of cognitive-based training in healthy older people, and help the healthy older people obtain the greatest possible benefit in health promotion and disease prevention.

## Introduction

The global population is rapidly aging, and this has become an important issue in many countries [**1**]. This increasing elderly population also have an impact on the form of social services, health care, policies, and benefits available to them. However, as one ages, various cognitive abilities decline; therefore, older individuals are unable to comprehend and respond to stimuli as fast as their younger counterparts [**2**]. Cognitive functions can be classified into four major domains on the basis of their characteristics: (1) Memory, involves the retention, storage, and recall of information. In the case of elderly individuals with normal cognitive functions, memory loss is strongly correlated with dementia [**3,4**].; (2) Attention, refers to the ability to quickly process information, and constrained with increasing age [**5,6**].; (3) Executive function is a multi-faceted construct that has been conceptualized from a variety of contexts, include the ability for planning, directing and maintaining attention, organization, abstract reasoning and problem-solving, self-regulation, and motor control, and refers to a range of higher-level cognitive processes [**7**].; (4) Visual-spatial ability, defined as the ability of the brain to respond to planar and three-dimensional spaces, and is considered to be age-related decline [**8**].

The goal of cognitive-based training for the older people is not to teach new developmental skills, but rather to maintain functions [**9**]. Cognitive-based training can be divided into three major categories: cognitive stimulation, cognitive training, and cognitive rehabilitation [**10**]. The majority studies in conducting cognitive-based training was in the frequency of ≧3 times each week [**11,12,13,14**], total training weeks of ≧8 weeks [**15,16,17**] and total training sessions of≧24 sessions [**15,16,18,19**]. Three previous studies have performed meta-analyses to address the effect of cognitive-based training in healthy older people individuals [**20,21,22**]. The gaps in previous studies were: (1) They have not done an independent investigation and analysis of the effects on healthy older people individuals alone; (2) The lack of multiple indicators of cognitive sub-functions (such as memory, attention, executive function, and visual-spatial ability); and (3) The past searches imposed restrictions on language and date of publication. Therefore, this study aim to investigate the effect of cognitive-based training on healthy older people individuals alone; in addition, this study sought to assess whether the characteristics of training had any impact on effect size.

## Material and methods

### Reporting standards

The current study was conducted and reported according to the Reporting Items for Systematic reviews and Meta-Analyses (PRISMA); [**23**]) statement for meta-analysis of randomized controlled tails (RCTs).

### Search strategy

We collected all available quantitative research of cognitive-based training on the healthy older people for meta-analysis. From Cochrane, PubMed, EMBASE, MEDLINE, PsycINFO, and CINAHL; no restrictions were placed on the publication year of the articles or the language. The search was conducted up to December 10, 2016. The keywords were specified by the Cochrane Library's Cochrane Dementia and Cognitive Improvement Group. The six cognitive-based training-related keywords: "cognitive-based training", "cognitive stimulation", "cognitive rehabilitation", "cognitive retraining", "cognitive re-training" and "cognitive support" were used when specifying a Medical Subject Heading (MeSH). We restricted the article type to RCT, age range >65 years; for the data-base that cannot set such restrictions (e.g. Cochrane), we add the key words: “RCT” and “elderly”, “aging” or “older” to confirm the comprehensive search strategy. The Google search was also used to locate any articles possibly missing from the foregoing databases.

### Selection criteria

Articles had to comply with the following inclusion and exclusion criteria: (1) Research design: The research design had to comply with the three criteria of randomization, control, and experimental implementation. (2) Intervention and control group: Intervention could consist of a single type or mixed types of cognitive-based training. Any form of control group was permissible, including groups receiving other treatment methods or a group on a waiting list. (3) Research participants: The research participants had to be older people individuals with normal cognitive functions, and have not diagnosed with mild cognitive impairment or any form of dementia. (4) Outcome indicators: This study emphasized variables connected with cognitive functions, including overall cognitive function, memory, attention, executive function, and visual-spatial ability. (5) Other criteria: Studies had to possess sufficient statistical data to enable the calculation of effect size.

### Outcome measures

This study classified outcome measures into five types based on the literature before assessing effectiveness: change in overall cognitive function, memory, attention, executive function, and visual-spatial ability [**24**]. The primary outcome is overall cognitive function; secondary outcomes are memory, attention, executive function, and visual-spatial ability.

### Data extraction

We use Cohen’s k to evaluate the reliability between raters and registrants to avoid bias associated with sample selection or variable interpretation, with a value of k> 0.65 indicating acceptable consistency between raters and registrants [**25**]. Two of this study's authors separately registered all of the selected RCTs with regard to design, diagnosis, intervention, and outcome variables, and both performed an inter-rater reliability test, the resulting Kappa value was 0.88. If the two authors had different opinions, then the third senior author reconciled the difference. If a study failed to include some required data within the article, we then contacted the authors directly to obtain the necessary information.

### Assessment of methodological quality

This study used the quality record guidelines proposed by the Cochrane Collaboration Guidelines [**26**] to assess five major forms of bias present in the RCTs: adequacy of sequence allocation, allocation concealment, blinding, incomplete outcome data, and selective reporting. The research quality of the study design, outcome measures, statistical analysis, and results of the selected RCTs were assessed using the approach with a total research-quality score of six to ten indicating an acceptable level of quality, and a score less than or equal to five indicating an unacceptable level of quality [**27**]. The resulting Kappa value for intra-rater reliability was 0.87.

### Data analysis

This study employed Comprehensive Meta-Analysis Version 2.0 to perform the meta-analysis. After the calculation of Hedges’ effect size, the magnitude of Hedges’ g may be interpreted using Cohen's convention as 0.2 comprised a small effect size, 0.5 comprised a moderate effect size, and 0.8 comprised a large effect size [**28**]. The Q value and I^2^ value were used to test for homogeneity and heterogeneity. Taking each quartile as an interval, a value equal to 0% indicated no heterogeneity, a value less than 25% indicated minor heterogeneity, a value of 50% indicated moderate heterogeneity, and a value of over 75% indicated a high degree of heterogeneity [**29**]. All overall effect size values in this study were inspected using the random effect model, because it accommodated the possibility that the underlying effect differed across studies. The random-effects model is more conservative and has a wider 95% CI than a fixed-effects model.

Subgroup analysis and meta-regression was further employed to determine which standards and characteristics were significant and had relatively large effects. Variables were examined using a mixed-effects model that was based on both the fixed-effects and random-effects models. In subgroup analysis, we used the QB test for between-groups comparison, a method similar to variance analysis. Sensitivity analysis was applied to determine whether overall effect size was affected by research quality, and Cochrane Collaboration Guideline scores were used to analyze differences in effect size. This study employed funnel plots and Egger's regression intercept to determine publication bias.

## Results

### Selection and characteristics of studies

**Fig 1:**
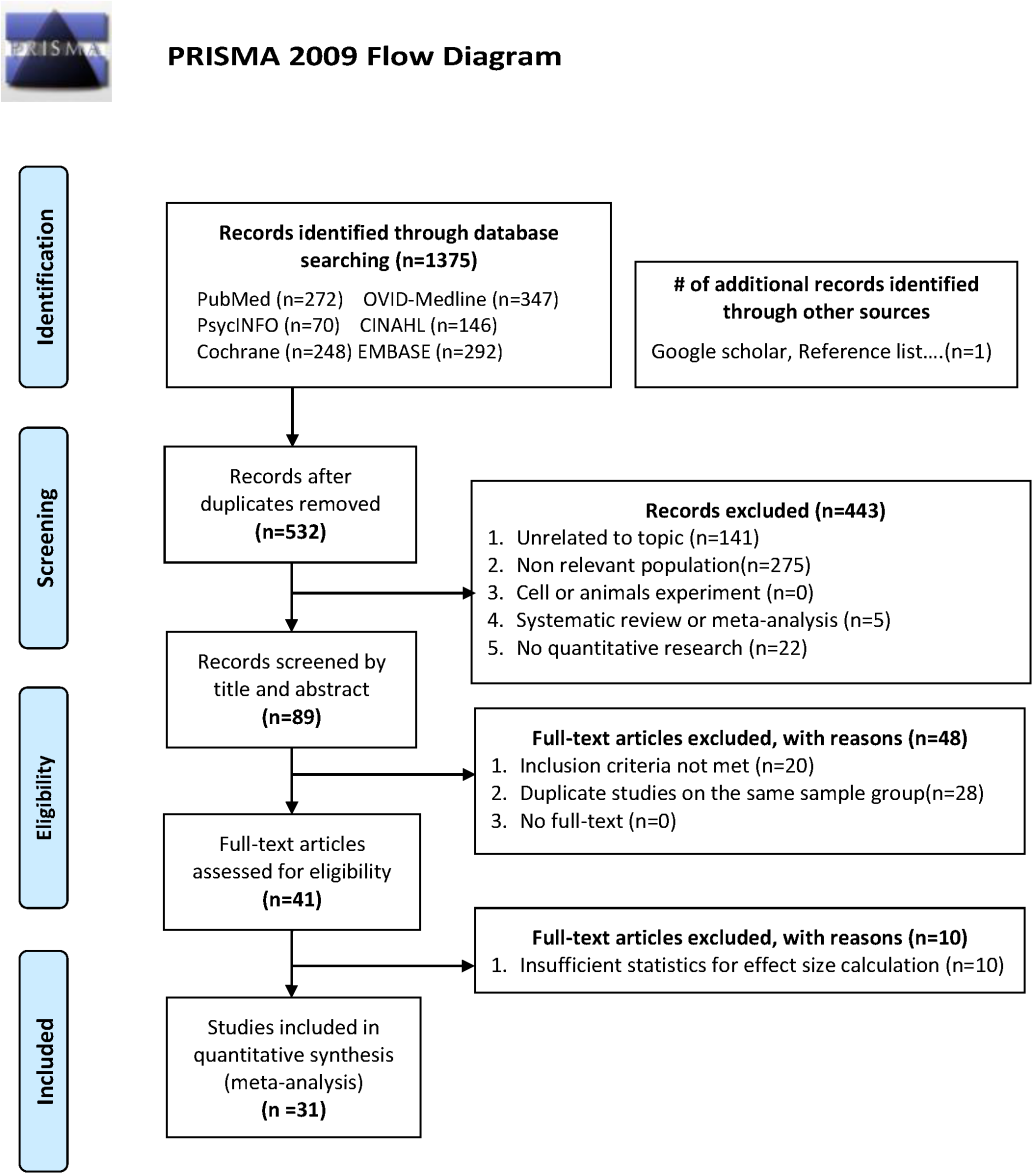
Study selection flow chart.

A total of 1,375 unique titles and abstracts were retrieved from the search (Fig 1). Ultimately, 31 RCTs met inclusion criteria for primary analysis. Among the 31 RCTs, 14 analyzed the effectiveness indicator of overall cognitive function, 20 analyzed memory, 20 analyzed attention, 22 analyzed executive function, and 6 analyzed visual-spatial ability (Table 1).

**Table 1:** Characteristics of included studies (n = 31).

Study citation (author/year)	Participants	Intervention characterization	Outcomes of interest/ Measurement tool	Follow-up time	Study Quality/ Cochrane tool
	• Age (mean age) • Gender (male/female) • Education (years) • Cognitive function	• EG/CG • Formant • Frequency			
Ball et al. /2002	• Total N: 2832 • Complete N (EG/CG): EG1 = 640, EG2 = 629, EG3 = 653/CG = 639 • Mean age: 73.6±5.9 (range:65–94) • Gender (M/F): 676/2126 • Education: 13.5 • Cognitive function (assessed by MMSE): total = 27.3±2.0 (range = 23–30)	• **EG1:** memory training • **EG2:** reasoning training • **EG3:** speed-of-processing training • **CG:** no contact • **Format:** group • **Frequency:** • 60-75min. a da • days of week: 2–3 • total weeks: 5–6 • total sessions: 10	• **Memory**: episodic verbal memory • **Attention**: useful field of view task, • **Executive function**: reasoning test	• Pre-treatment • Post-treatment • 1 year evaluation • 2 year evaluation	• 10/ • AA: low • AC: unclear • BAO: low(single) • IO: high • SRO: low
Ballesteros et al. /2015	• Total N: 40 • Complete N (E/C): EG = 17/CG = 13 • Mean age: EG = 68.5/CG = 69.2 (range: 57–80) • Gender (M/F): 12/18 • Education: EG = 12.2±5.09/CG = 12.9±3.28 • Cognitive function (assessed by MMSE): EG = 28.7±1.16/CG = 28.8±1.03	• **EG:** brain training with non-action video games • **CG:** education control • **Format:** individual • **Frequency:** • min. a day: 60 • days of week: 1–2 • total weeks: 10–12 • total sessions: 20	• **Memory**: wechsler memory scale-III (WMS-III) • **Attention**: cross-modal oddball attention tas • **Executive function**: wisconsin card sort test (WCST)	• Pre-treatment • Post-treatment • 3 month evaluation	• 8/ • AA: low • AC: unclear • BAO: low(single) • IO: low • SRO: low
Basak et al. /2008	• Total N: 40 • Complete N (E/C): EG = 19/CG = 20 • Mean age: EG = 70.05±4.94/CG = 69.10±6.06 • Gender (M/F): 10/29 • Education: EG = 15.42±3.49/CG = 16.88±3.18 • Cognitive function (assessed by modified MMSE): EG = 55.68±1.80/CG = 55.65±1.39	• **EG:** real-time strategy videogame (RON) • **CG:** no contact • **Format:** individual • **Frequency:** • min. a day: 90 • days of week: 3–4 • total weeks:4–5 • total sessions: 15	• **Memory**: visual short term memory, N-back • **Attention**: stopping task, attentional blink, functional field of view • **Executive function**: operation span, task switching, matrices • **Visuo-spatial ability**: enumeration, mental rotation	• Pre-treatment • Post-treatment	• 5/ • AA: low • AC: unclear • BAO: unclear • IO: low • SRO: low
Blackwood et al. /2016	• Total N: 44 • Complete N (E/C): EG = 19/CG = 25 • Mean age: EG = 73.79±4.36/CG = 75.68±6.88 (range: >65) • Gender (M/F): 12 /32 Cognitive function (assessed by MoCA): EG = 25.26±2.33/CG = 24.40±3.22	• **EG:** home-based computerized cognitive training • **CG:** usual care • **Format:** individual • **Frequency:** • min. a day: 20–25 • days of week: 3 • total weeks: 6 • total sessions: 18	• **Cognitive function**: montreal cognitive assessment (MoCA) • **Executive function**: trial making test-B (TMT-B)	• Pre-treatment • Post-treatment	• 9/ • AA: low • AC: unclear • BAO: low(single) • IO: low • SRO: low
Bozoki et al. /2013	• Total N: 60 • Complete N (E/C): EG = 32/CG = 28 • Mean age: 68.9±6.76 (range: >60) • Gender (M/F): 25/35 • Education: 16.7/16.9 • Cognitive function (assessed by SLUMS): total = 27.2±2.16 (range>22)	• **EG:** real-time strategy videogame (RON) • **CG:** no contact • **Format:** individual • **Frequency:** • min. a day: 90 • days of week: 3–4 • total weeks:4–5 • total sessions: 15	• **Memory**: visual short term memory, N-back • **Attention**: stopping task, attentional blink, functional field of view • **Executive function**: operation span, task switching, matrices • **Visuo-spatial ability**: enumeration, mental rotation	• Pre-treatment • Post-treatment	• 5/ • AA: low • AC: unclear • BAO: unclear • IO: low • SRO: low
Cheng et al. /2012	• Total N: 270 • Complete N (E/C): EG1 = 54, EG2 = 59 /CG = 60 • Mean age: EG1 = 70.79±3.33, EG2 = 69.78±3.76/CG = 70.17±3.47 (range: 65–75) • Gender (M/F): 138/132 • Education: EG1 = 9.33±3.8, EG2 = 9.77±3.96 /CG = 9.58±3.93 • Cognitive function (assessed by MMSE): EG1 = 27.16±2.13/ EG2 = 27.33±2.14/ CG = 26.86±2.22 (range>19)	• **EG1:** multi-domain cognitive training • **EG2:** singe-domain cognitive training • **CG:** waiting-list • **Format:** group • **Frequency:** • min. a day: 60 • days of week: 2 • total weeks: 12 • total sessions: 24	• **Cognitive function**: repeatable battery for the assessment of neuropsychological status (RBANS) • **Memory**: RBANS-immediate memory, delayed memory • **Attention**: color word stroop test (CWST) • **Executive function**: visual reasoning test • **Visuo-spatial ability**: RBANS-visuospatial	• Pre-treatment • Post-treatment • 6 month evaluation • 1 year evaluation	• 9/ • AA: low • AC: low • BAO: low(double) • IO: low(ITT) • SRO: low
Craik et al. /2007	• Total N: 49 • Complete N (E/C): EG = 29/CG = 20 • Mean age: 78.7±3.9 (range:71–87) • Gender (M/F): 22/27 • Education: EG = 14.36±3.34/CG = 14.55±4.71 • Cognitive function (assessed by MMSE)	• **EG:** cognitive rehabilitation training • **CG:** late-training • **Format:** group • **Frequency:** • min. a day: 180 • days of week: 1 • total weeks: 12 • total sessions: 12	• **Memory**: hopkins verbal learning test-rivised (HVLT-R), brown peterson • **Executive function**: alpha span	• Pre-treatment • Post-treatment • 6 month evaluation	• 7/ • AA: low • AC: unclear • BAO: unclear • IO: low • SRO: low
Edwards et al. /2015	• Total N: 73 • Complete N (E/C): EG = 27/CG = 33 • Mean age: 73.99 ±7.48 (range:59–95) • Gender (M/F): 21/46 • Education: EG = 16.04±2.21/CG = 16.00±2.22 • Cognitive function (assessed by MMSE): EG = 28.37±1.57/CG = 27.85±1.87 (range>23)	• **EG:** cognitive speed of processing training (SOPT) • **CG:** waiting list • **Format:** individual • **Frequency:** • min. a day: 60 • days of week: 2–3 • total weeks: 10–12 • total sessions: 20	• **Attention:** usuful field of view test	• Pre-treatment • Post-treatment	• 6/ • AA: low • AC: unclear • BAO: unclear • IO: low • SRO: unclear
Garcia-Campuzano et al. /2014	• Total N: 24 • Complete N (E/C): EG = 13/CG = 11 • Mean age: EG = 78.43/CG = 75.18 • Gender (M/F): 5/19	• **EG:** computerized cognitive training • **CG:** late-training • **Format:** individual • **Frequency:** • min. a day: 30 • days of week: 3 • total weeks: 8 • total sessions: 24	• **Memory**: wechsler memory scale (WMS)	• Pre-treatment • Post-treatment	• 5/ • AA: low • AC: unclear • BAO: unclear • IO: low • SRO: unclear
Kawashima /2013	• Total N: 124 • Complete N (E/C): EG = 51/CG = 47 • Mean age: 85.1 ± 5.4 (range:76–96) • Gender (M/F): 54 /44 • Education: EG = 12.9 ±3.9/CG = 12.5 ±3.3 • Cognitive function (assessed by MMSE): range >23	• **EG:** learning therapy • **CG:** usual care **Format:** individual **Frequency:** • min. a day: 15–20 • days of week: 5 • total weeks: 24 • total sessions: 120	• **Cognitive function:** MMSE • **Attention:** digit symbol • **Executive function:** Frontal Assessment Battery (FAB)	• Pre-treatment • Post-treatment	• 9/ • AA: low • AC: low • BAO: low(single) • IO: low • SRO: unclear
Kim et al. /2015	• Total N:85 • Complete N (E/C): EG1 = 24, EG2 = 24/CG = 37 • Mean age: EG1 = 68.0±6.1, EG2 = 67.7±5.4 /CG = 66.9±4.0 (range: >60) • Gender (M/F): 60/25 • Education: EG1 = 13.2±3.9, EG2 = 14.0±3.3 /CG = 13.2±3.7 • Cognitive function (assessed by K-MMSE): EG1 = 28.9±1.5, EG2 = 29.1±0.9/CG = 13.2±3.7 (range >26)	• **EG1:** robot-assisted multi-domain cognitive training • **EG2:** traditional multi-domain cognitive training • **CG:** usual care • **Format:** group • **Frequency:** • min. a day: 90 • days of week 5 • total weeks: 12 • total sessions: 60	• **Cognitive function:** the alzheimer's disease assessment scale-cognitive subscale **(**ADAS-cog) • **Memory:** delayed matching to sample, pattern recognition memory, paired associate learning, spatial working memory • **Attention:** rapid visual information processing, reaction time • **Executive function:** stocking of cambridge	• Pre-treatment • Post-treatment	• 10/ • AA: low • AC: unclear • BAO: low(double) • IO: low • SRO: low
Kwok et al. /2013	• Total N: 200 • Complete N (E/C): EG = 86/CG = 90 • Mean age: 75.41 ± 7.31 (range: >60) • Gender (M/F): 30/170 • Education: total = 3.56 • Cognitive function (assessed by Cantonese version of MMSE): EG = 25.74±2.27/ CG = 25.92±2.30 (range >23)	• **EG:** cognitive Training • **CG:** usual care • **Format:** group • **Frequency:** • min. a day: 60 • days of week 1 • total weeks: 8 • total sessions: 8	• **Cognitive function:** clinical dementia rating scale (CDRS) • **Memory:** CDRS-memory • **Attention:** CDRS-attention • **Executive function:** CDRS-initiation/preservation	• Pre-treatment • Post-treatment	• 9/ • AA: low • AC: unclear • BAO: unclear • IO: low • SRO: low
Lee et al. /2013	• Total N: 35 • Complete N (E/C): EG = 15/CG = 16 • Mean age: 65.1 ± 2.9 (range: 60–70) • Gender (M/F): 14/21 • Cognitive function (assessed by MMSE): total = 28.3±1.3 (range >26)	• **EG:** brain-computer interface based cognitive training • **CG:** waiting-list • **Format:** individual • **Frequency:** • min. a day: 30 • days of week: 3 • total weeks: 8 • total sessions: 24	• **Cognitive function:** repeatable battery for the assessment of neuropsychological status (RBANS) • **Memory**: RBANS-immediate memory, RBANS-delayed memory • **Attention:** RBANS-attenrion • **Visuo-spatial ability:** RBANS-visuospatial	• Pre-treatment • Post-treatment • 2 month evaluation	• 9/ • AA: low • AC: low • BAO: unclear • IO: low • SRO: low
Lee et al. /2015	• Total N: 39 • Complete N (E/C): EG = 21/CG = 18 • Mean age: 65.2 ± 2.8 (range: 60–70) • Gender (M/F): 27/12 • Cognitive function(assessed by MMSE): total = 27.6±1.6 (range≧26)	• **EG:** brain-computer interface cognitive training program • **CG:** waiting-list • **Format:** individual • **Frequency:** • min. a day: 30 • days of week: 3 • total weeks: 8 • total sessions: 24	• **Cognitive function:** RBANS • **Memory**: RBANS-immediate memory, RBANS-delayed memory • **Attention:** RBANS-attenrion • **Visuo-spatial ability:** RBANS-visuospatial	• Pre-treatment • Post-treatment	• 9/ • AA: low • AC: unclear • BAO: high • IO: low • SRO: low
Legault et al. /2011	• Total N: 73 • Complete N(E/C): EG1 = 16, EG2 = 16, EG3 = 18/CG = 17 • Mean age: 76.4 (range:70–85) • Gender (M/F): 34/39 • Cognitive function (assessed by modified MMSE): EG = 55.68±1.80/CG = 55.65±1.39	• **EG1:**cognitive training • **EG2:**physical activity training • **EG3:**combined intervention • **CG:** education • **Format:** group • **Frequency:** • min. a day: 40–48 • days of week: 1–2 • total weeks: 16 • total sessions: 24	• **Memory:** hopkins verbal learning test (HVLT), logical memory task • **Executive function:** the self-ordered pointing task, 1-back and 2-back Tests, the eriksen flanker task, the task switching test, trial making test (TMT)	• Pre-treatment • Post-treatment	• 8/ • AA: low • AC: unclear • BAO: low(single) • IO: low(ITT) • SRO: low
Linde et al. /2014	• Total N: 70 • Complete N (E/C): EG1 = 18, EG2 = 19, EG3 = • 17/CG = 16 • Mean age: EG1 = 68.28±2.02, EG2 = 67.63±4.40, EG3 = 65.59±3.74 /CG = 66.56±3.20 (range: 60–75) • Gender (M/F): 29/41	• **EG1:**cognitive activity intervention • **EG2**:physical activity intervention • **EG3**:combined intervention • **CG:** waiting-list • **Format:** group • **Frequency:** • min. a day: 30 • days of week: 1 • total weeks: 16 • total sessions: 16	• **Memory**: word list test • **Attention:** trail making test-A, digit-symbol, d2 • **Executive function:** reasoning test (performance test system, LPS 50+) • **Visuo-spatial ability:** spatial relation (subset of LPS 50+)	• Pre-treatment • Post-treatment • 4 month evaluation	• 9/ • AA: low • AC: high • BAO: low(single) • IO: low • SRO: low
Mahncke et al. /2006	• Total N: 182 • Complete N (E/C):EG = 53/CG1 = 53,CG2 = 56 • Mean age: 70.9 (range:60–87) • Gender (M/F): 91/91 • Education: 16.3 • Cognitive function (assessed by MMSE): range>24	• **EG:** brain plasticity-based training program • **CG1:** active control • **CG2:** non-contact control • **Format:** individual • **Frequency:** • min. a day: 60 • days of week: 5 • total weeks: 8–10 • total sessions: 40	• **Cognitive function:** repeatable battery for the assessment of neuropsychological status (RBANS) • **Memory:** global auditory memory • **Executive function:** digit span	• Pre-treatment • Post-treatment • 3 month evaluation	• 7/ • AA: low • AC: unclear • BAO: low(double) • IO: low • SRO: low
Margrett et al. /2006	• Total N: 106 • Complete N (E/C): EG1 = 30, EG2 = 34 /CG = 34 • Mean age: 71.43 ± 5.85 (range: 61–89) • Gender (M/F): 53/53 Education: 15.95 ± 3.06	• **EG1:**inductive reasoning training program (individual) • **EG2:** inductive reasoning training program (collaborative) • **CG:** no-treatment • **Format:** individual • **Frequency:** • min. a day: 60–75 • days of week: 2–3 • total weeks: 5–6 • total sessions: 10	• **Executive function:** reasoning test (letter series test, word series test, letter sets test)	• Pre-treatment • Post-treatment	• 7/ • AA: low • AC: unclear • BAO: unclear • IO: low • SRO: low
Millán-Calenti et al. /2015	• Total N: 160 • Complete N (E/C): EG = 80 /CG = 80 • Mean age: 74.34 ± 6.40 (range:≧65) • Gender (M/F): 36/106 • Cognitive function (assessed by MMSE): 27.76 ±1.75 (range>24)	• **EG:** computerized cognitive training • **CG:** no-treatment • **Format:** individual • **Frequency:** • min. a day: 20 • days of week: depends on participant • total weeks: 12 • total sessions: depends on participant	• **Cognitive function:** MMSE	• Pre-treatment • Post-treatment	• 8/ • AA: low • AC: unclear • BAO: unclear • IO: low • SRO: low
Mozolic et al. /2011	• Total N: 66 • Complete N (E/C): EG = 30/CG = 32 • Mean age: 69.4 ± 3.2 (range:65–75) • Gender (M/F): 31/35 • Education: EG = 15.6 ±2.2/CG = 16±3.4 • Cognitive function (assessed by MMSE): EG = 28.3±1.5/CG = 28.5±1.9	• **EG:** visual and auditory tasks • **CG:** educational lecture • **Format:** individual • **Frequency:** • min. a day: 60 • days of week: 1 • total weeks: 8 • total sessions: 8	• **Memory:** Hopking verbal learning (HVLT), N-back • **Attention:**selective attention, divided attention, selective auditory attention, selective visual attention, digit symbol	• Pre-treatment • Post-treatment	• 7/ • AA: low • AC: low • BAO: low(single) • IO: low • SRO: low
Nouchi et al. /2012	• Total N: 32 • Complete N (E/C): EG = 14/CG = 14 • Mean age: EG = 68.86 ±2.07 /CG = 68.31 ±2.82 (range:>65) • Gender (M/F):13/15 • Education: EG = 13.43±2.38/CG = 13.36±2.13 • Cognitive function (assessed by MMSE): EG = 28.50±1.16/CG = 28.50±1.51 (range>26)	• **EG:** brain training game • **CG:** active control • **Format:** individual • **Frequency:** • min. a day: 15 • days of week: 5 • total weeks: 4 • total sessions: 20	• **Cognitive function:** MMSE • **Attention:** digit cancellation task (D-CAT), digit symbol coding, digit symbol search • **Executive function:** frontal assessment battery (FAB), TMT-B, digit span test	• Pre-treatment • Post-treatment	• 8/ • AA: low • AC: low • BAO: low(double) • IO: low • SRO: low
Park et al. /2014	• Total N: 40 • Complete N (E/C): EG = 20/CG = 20 • Mean age: 69.7 (range:>65) • Gender (M/F): 13 /27 • Education: 10.9 ±4.6/10.9 ±4.2 • Cognitive function (assessed by MMSE): EG = 29.3±1.4/CG = 28.88±1.5	• **EG:** transitional direct cognitive stimulation (tDCG) • **CG:** sham tDCG • **Format:** individua**l** • **Frequency:** • min. a day: 30 • days of week: 5 • total weeks: 2 • total sessions: 10	• **Memory:** two-back verbal working memory task • **Executive function:** digit span test	• Pre-treatment • 1 wk evaluation • 4 wk evaluation	• 7/ • AA: low • AC: unclear • BAO: low(double) • IO: low • SRO: low
Shatil et al. /2013	• Total N: 125 • Complete N (E/C):EG1 = 33, EG2 = 31, EG3 = 29 /CG = 29 • Mean age: 76.83±5.51 (range:>65) • Gender (M/F): 38/84 • Education: 15.7±2.43 • Cognitive function (assessed by MMSE): range >23	• **EG1:** cognitive intervention • **EG2:** physical activity intervention • **EG3:** combined cognitive-physical activity intervention • **CG:** reading book and discussion • **Format:** group • **Frequency:** • min. a day: 40 • days of week: 3–4 • total weeks: 16 • total sessions: 48	• **Memory:** global visual memory, auditory working memory • **Attention:** divided attention	• Pre-treatment • Post-treatment	• 8/ • AA: low • AC: unclear • BAO: unclear • IO: low • SRO: low
Shatil et al. /2014	• Total N: 119 Complete N (E/C):EG = 60 /CG = 59 • Mean age: EG = 67.7±5.8/ CG = 68.3±5.8 (range:60–87) • Gender (M/F): 44/75 • Education: 15.7±2.43 • Cognitive function (assessed by MMSE): EG = 28.4±2.1/CG = 28.9±1.4 (range≧27)	• **EG:** interactive television-based cognitive training • **CG:** active control • **Format:** individual • **Frequency:** • min. a day: 20 • days of week: 3 • total weeks: 8 • total sessions: 24	• **Attention:** trial making test-A • **Executive function:** digit span, trial making test-B	• Pre-treatment • Post-treatment	• 9/ • AA: low • AC: unclear • BAO: low(single) • IO: low • SRO: low
Smith et al. /2009	• Total N: 487 • Complete N (E/C): EG = 242 /CG = 245 • Mean age: EG = 75.6 ± 6.6 /CG = 75.0± 6.3 (range:>65) • Gender (M/F): 232 /255 • Education: EG = 15.7±2.6 /CG = 15.6±2.6 • Cognitive function (assessed by MMSE): EG = 29.1±1.1/CG = 29.2±1.0 (range >26)	• **EG:** computerized cognitive training program • **CG:** active control • **Format:** individual • **Frequency:** • min. a day: 60 • days of week: 5 • total weeks: 8 • total sessions: 40	• **Cognitive function:** repeatable battery for the assessment of neuropsychological status (RBANS) • **Memory:** neuropsychological overall memory, rivermead behavioral memory test, wechsler memory scale (WMS) • **Attention:** processing speed • **Executive function:** digit span	• Pre-treatment • Post-treatment	• 10/ • AA: low • AC: low • BAO: low(double) • IO: low(ITT) • SRO: low
Song et al. /2009	• Total N: 129 • Complete N (E/C): EG = 74 /CG = 55 • Mean age: 78.23± 6.22 (range:>65) • Gender (M/F): 9 /120 • Education: 4.62± 4.33 • Cognitive function (assessed by Korean version of MMSE)	• **EG:** cognitive training • **CG:** late-training • **Format:** group • **Frequency:** • min. a day: 60 • days of week: 2 • total weeks: 12 • total sessions: 12	• **Cognitive function:** MMSE	• Pre-treatment • 8 wk evaluation • 16 wk evaluation • 24 wk evaluation	• 8/ • AA: low • AC: unclear • BAO: unclear • IO: low • SRO: low
Stine-Morrow et al. /2008	• Total N: 181 • Complete N (E/C): EG = 87/CG = 63 • Mean age: EG = 73.0/CG = 72.0 • Education: EG = 16.3/CG = 16.0	• **EG:** odyssey of the mind program • **CG:** waiting-list • **Format:** group • **Frequency:** • min. a day: 60 • days of week: 1 • total weeks: 20 • total sessions: 20	• **Attention:** letter and pattern comparison tasks • **Executive function:** letter-number sequencing, everyday problem solving • **Visuo-spatial ability:** card rotation and hidden patterns	• Pre-treatment • Post-treatment	• 6/ • AA: low • AC: high • BAO: unclear • IO: low • SRO: low
Suzuki et al. /2014	• Total N: 58 • Complete N (E/C): EG = 29/CG = 29 • Mean age: EG = 73.0±7.1/CG = 73.3±5.4 (range:>65) • Gender (M/F): 5/53 • Education: EG = 12.6±2.0/CG = 13.1±2.5 • Cognitive function (assessed by MMSE): EG = 27.1±1.7/CG = 26.6±2.2	• **EG:** cognitive intervention • **CG:** education control • **Format:** group • **Frequency:** • min. a day: 120 • days of week: 1 • total weeks: 12 • total sessions: 12	• **Cognitive function:**MMSE, MoCA-J • **Memory:** logical memory, • **Attention:** trial making test-A • **Executive function:** digit span, trial making test-B, verbal fluency tests	• Pre-treatment • Post-treatment	• 8/ • AA: low • AC: unclear • BAO: low(single) • IO: low • SRO: low
Talib et al. /2008	• Total N: 23 • Complete N (E/C): EG = 11/CG = 12 • Mean age: EG = 67.82 ± 4.37/CG = 67.25 ± 4.09 • Education: EG = 14.64 ±2.24/CG = 16.67 ±3.02 • Cognitive function (assessed by MMSE): EG = 28.5±1.7/CG = 29.3±0.6	• **EG:** cognitive training • **CG:** usual care • **Format:** group • **Frequency:** • min. a day: 90 • days of week: 2 • total weeks: 2 • total sessions: 4	• **Memory:** prose recall, list recall • **Attention:** digit symbol • **Executive function:** categorization	• Pre-treatment • Post-treatment	• 7/ • AA: low • AC: high • BAO: unclear • IO: low • SRO: low
Wolinsky et al. /2013	• Total N: 207 • Complete N (E/C): EG1 = 45, EG2 = 44, EG3 = 60/CG = 58 • Mean age: EG1 = 72.1, EG2 = 71.0, EG3 = 70.7/CG = 72.0 (range: >65) • Gender (M/F): 94/113	• **EG1:** on-site visual speed of processing (VSP) training • **EG2:** on-site VSP training with booster • **EG3:** on-site VSP training at home • **CG:** attention control • **Format:** group • **Frequency:** • min. a day: 60–75 • days of week: 2–3 • total weeks: 5–6 • total sessions: 10	• **Attention:** useful field of view (UFOV), trail making test-A(TMT-A), digit symbol, stroop color test, digit vigilance test • **Executive function:** TMT-B, verbal fluency (controlled oral word association test)	• Pre-treatment • Post-treatment • 1 year evaluation	• 8/ • AA: low • AC: low • BAO: low(double) • IO: low • SRO: low
Yoon Mi et al. /2013	• Total N: 30 • Complete N (E/C): EG = 15/CG = 15 • Mean age: EG = 72.8 ± 3.8/CG = 71.7 ±5.6 (range: 65–80) • Gender (M/F): 13 /17 • Cognitive function (assessed by Korean version of MMSE): EG = 26.0±2.2/ CG = 26.1±1.6	• **EG:** computer-assisted cognitive rehabilitation training • **CG:** usual care • **Format:** individual • **Frequency:** • min. a day: 30 • days of week: 3 • total weeks: 6 • total sessions: 18	• **Cognitive function:** MMSE	• Pre-treatment • Post-treatment	• 5/ • AA: low • AC: unclear • BAO: unclear • IO: low • SRO: low

*Note*: EG, Experimental group; CG, Control group; min., minute; Cochrane tool: AA, adequacy of sequence allocation; AC, allocation concealment; BAO, blinding of assessors and outcomes; IO, incomplete outcome data; SRO, selective reporting and other biases

### Effect on overall cognitive function, memory, attention, executive function, and visual-spatial ability

**Fig 2:**
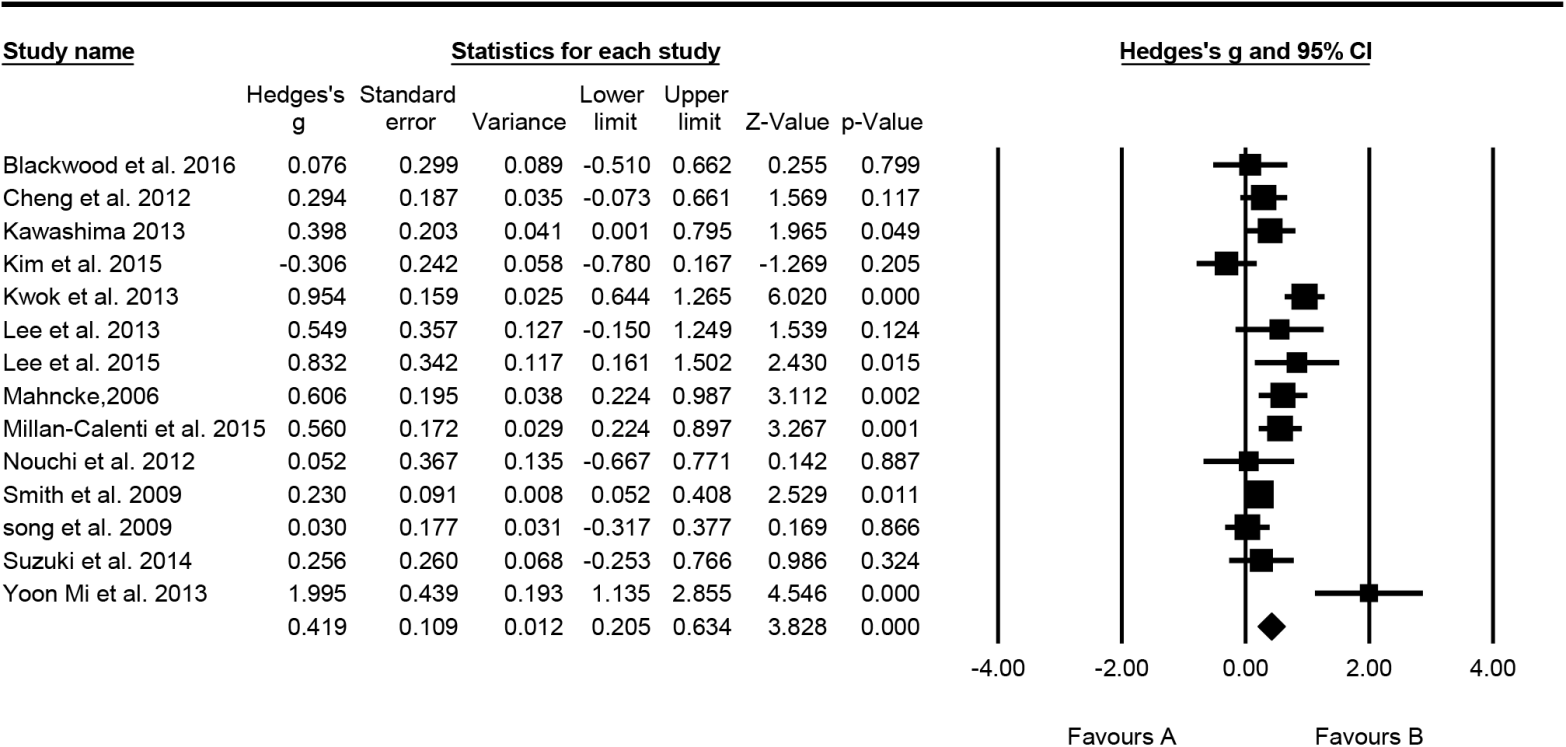
Effect of cognitive-based training on overall cognitive function (n = 14).

For overall cognitive function, 14 studies were included in the analysis; overall effect size expressed by Hedge's g = 0.419, the 95% confidence interval (CI) was 0.205–0.634, and the effect size directionality of the sample was positive. This indicated that cognitive-based training for healthy older people had a significant and moderate effect on overall cognitive function. The results of homogeneity testing showed that the Q value = 48.082, p = 0.000, I^2^ = 72.963%. The testing for publication bias indicated that the funnel plot was generally symmetrical, while the value of p = 0.425 for Egger's regression intercept indicated that publication bias was not significant (Fig 2).

**Fig 3:**
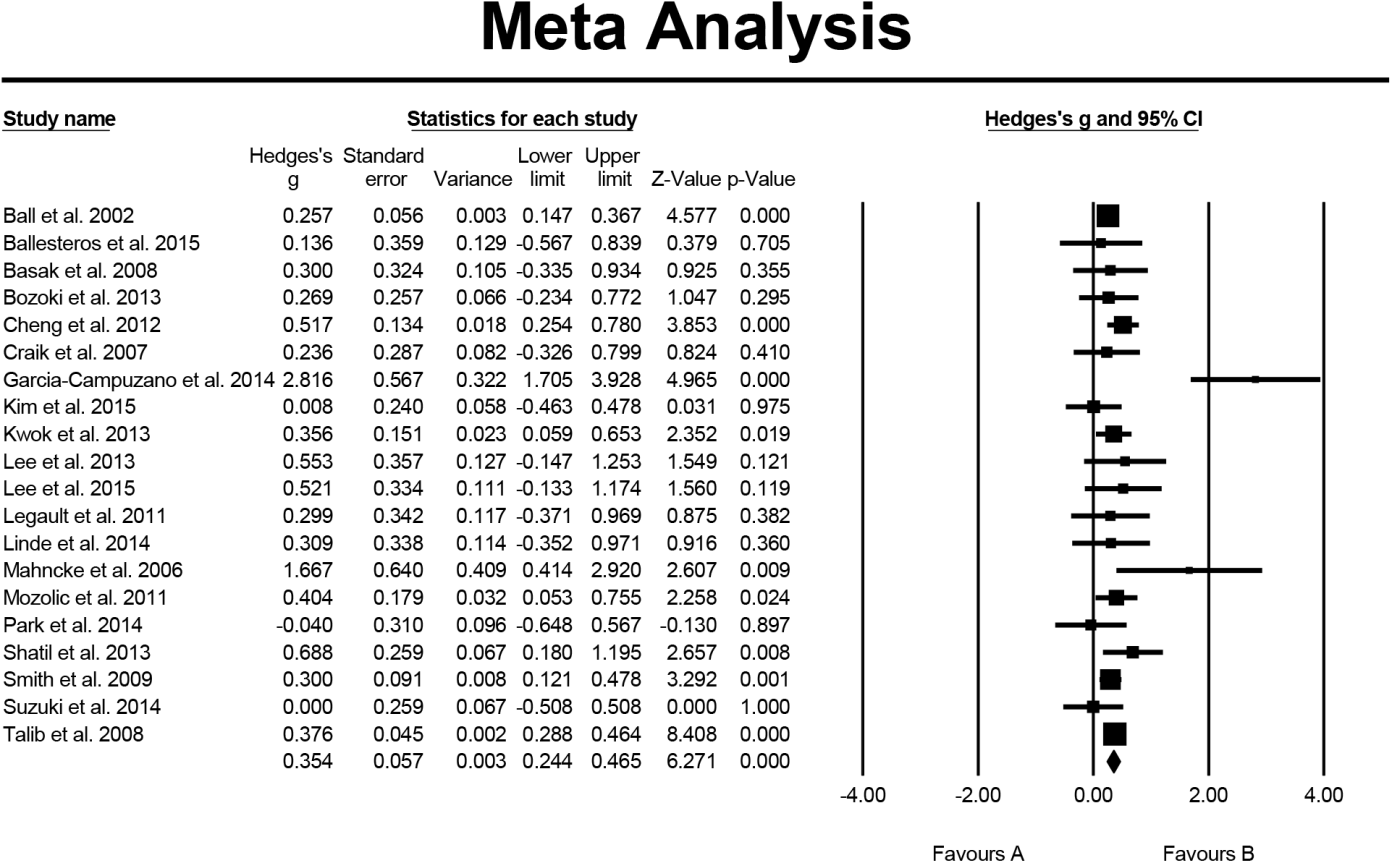
Effect of cognitive-based training on memory (n = 20).

Regarding to memory, 20 studies were included in the analysis; the overall effect size expressed by Hedge's g = 0.354, the 95% CI was 0.244–0.465, and the effect size directionality of the sample was positive. This indicated that cognitive-based training had a significant and small effect on the elders’ memory. The results of homogeneity testing showed that the Q value = 36.451, p = 0.009, I^2^ = 47.875%. The testing for publication bias indicated that the funnel plot was generally symmetrical; the value of p = 0.329 for Egger's regression intercept indicated that publication bias was not significant (Fig 3).

**Fig 4:**
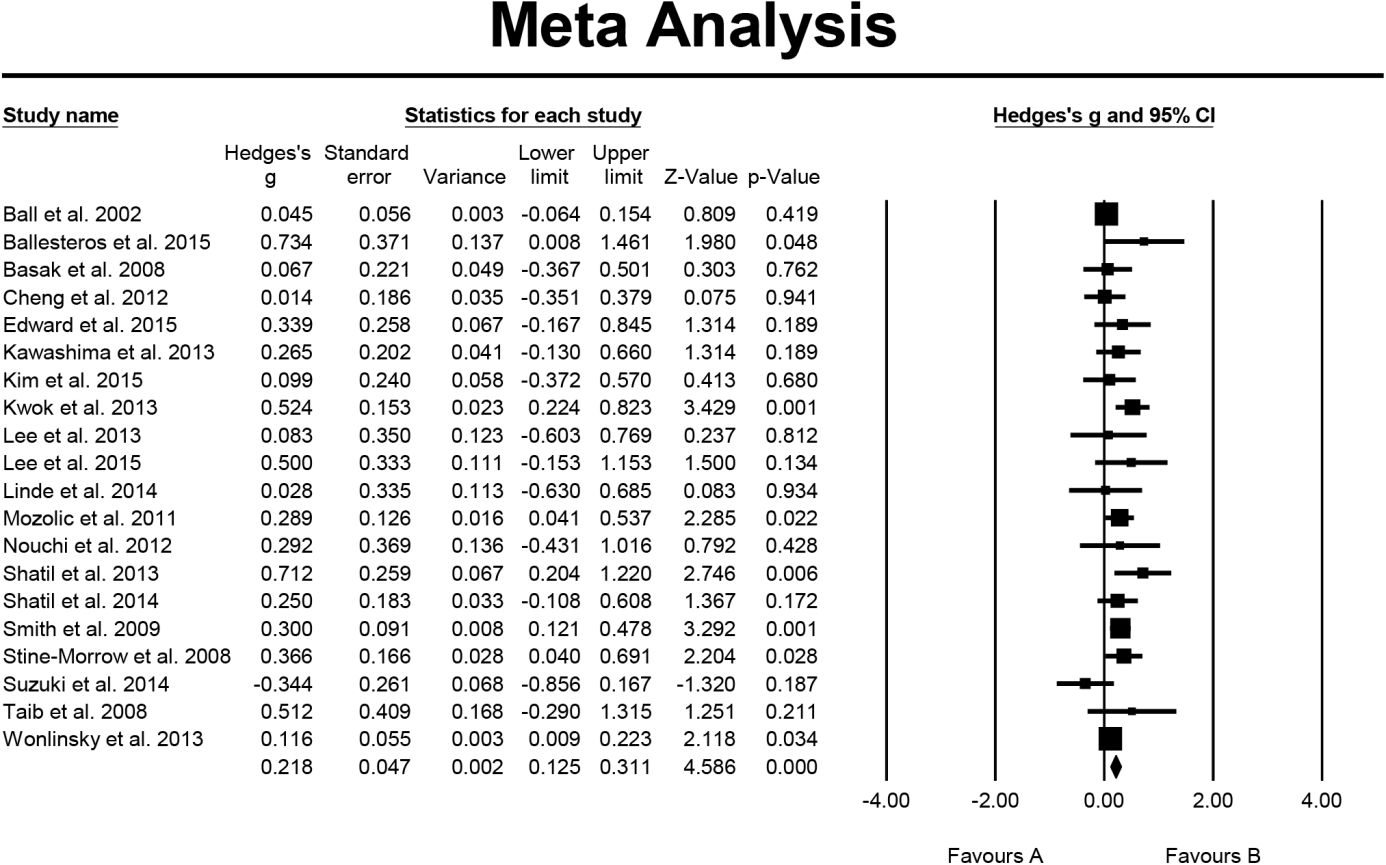
Effect of cognitive-based training on attention (n = 20).

On the study of attention, 20 studies were included in the analysis; the overall effect size expressed by Hedge's g = 0.218, the 95% CI was 0.125–0.311, the effect size directionality of the sample was positive. This indicated that the effect of cognitive-based training on attention was significant with a small effect size. The results of homogeneity testing showed that the Q value = 30.113, p = 0.050, I^2^ = 36.904%. The testing for publication bias indicated that the funnel plot was generally symmetrical; the value of p = 0.061 for Egger's regression intercept indicated that publication bias was not significant (Fig 4).

**Fig 5:**
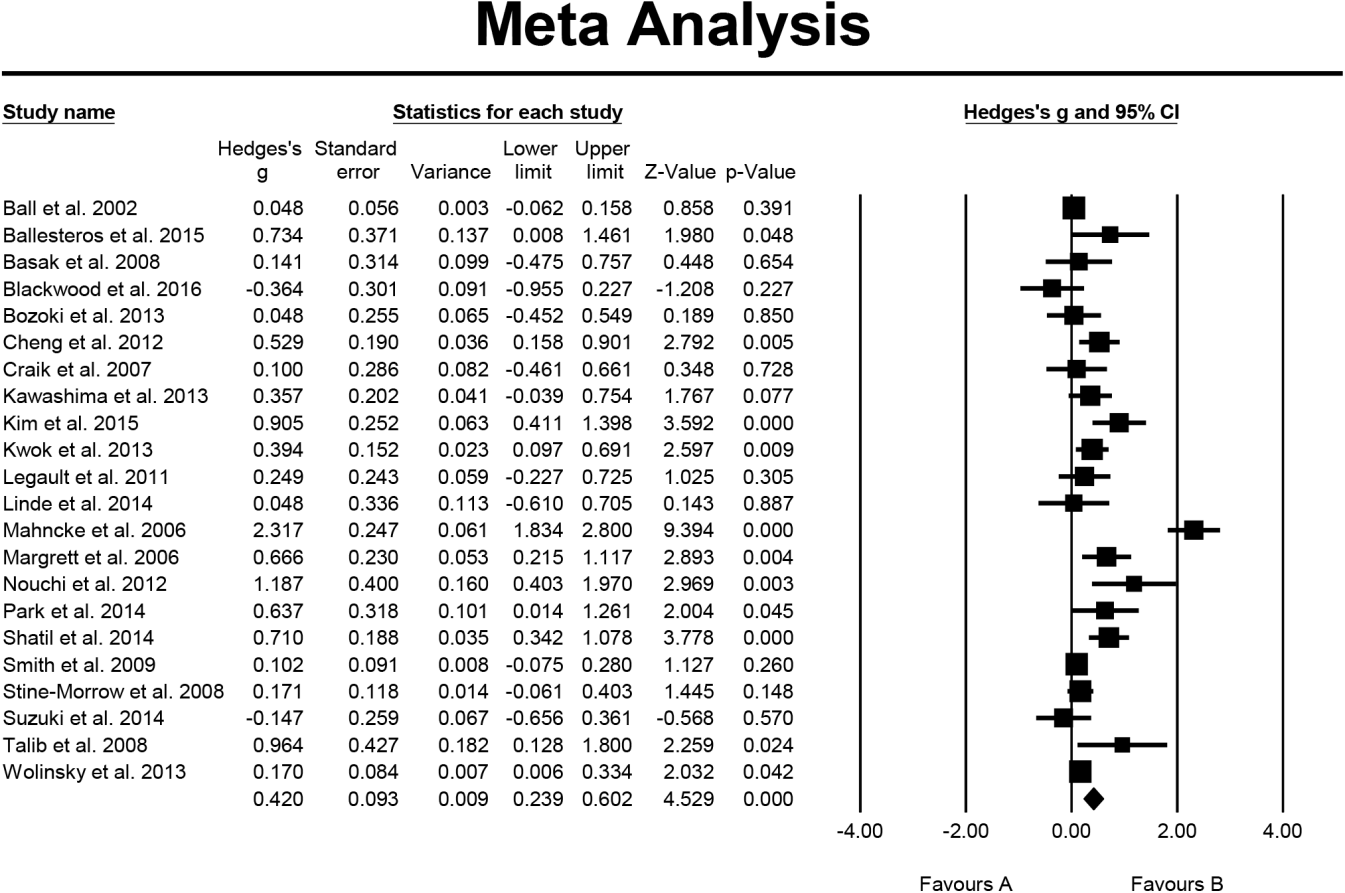
Effect of cognitive-based training on executive function (n = 22).

On the study of executive function, 22 studies were included in the analysis, the overall effect size expressed by Hedge's g = 0.420, the 95% CI was 0.239–0.601, the effect size directionality of the sample was positive. This indicated that cognitive-based training for healthy older people had a significant and moderate effect on executive function. The results of homogeneity testing showed that the Q value = 125.561, p = 0.000, I^2^ = 83.275%. The testing for publication bias indicated that the funnel plot was generally symmetrical; the value of p = 0.018 for Egger's regression intercept, and p = 0.185 for Begg’s test (Fig 5).

**Fig 6:**
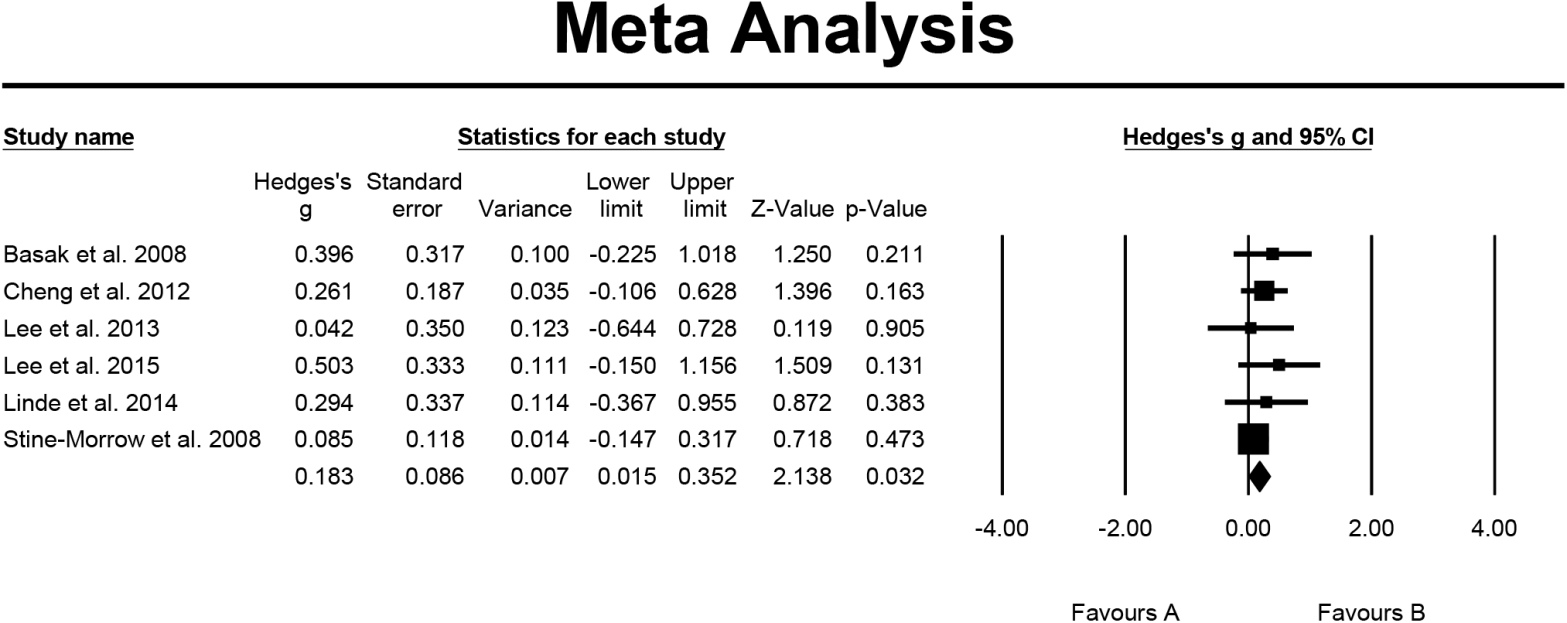
Effect of cognitive-based training on visual-spatial ability (n = 6).

For visual-spatial ability, six studies were included in the analysis, the overall effect size expressed by Hedge's g = 0.183 and the 95% CI was 0.015–0.352. This indicated that cognitive-based training has a significant and small effect on elders’ visual-spatial ability. The results of homogeneity testing showed that the Q value was not significant (Q = 2.505, p = 0.776, I^2^ = 0.000%), which indicated that the sample and research effect size were homogeneous. Further testing for publication bias indicated that the funnel plot was generally symmetrical, and the p = 0.136 for Egger's regression intercept indicated that publication bias was not significant (Fig 6).

The sensitivity analysis showed removal of any one study among the collected studies would not have affected the research results as a whole for all outcomes. This results supported the robustness of all the above results of the meta-analysis.

### Subgroup analysis of the characteristics of interventions

In this study, subgroup analysis were performed on the intervention characteristics in cognitive-based training. The subgroup analysis of the attention improvement revealed that the total number of training weeks ≧8 week is better than that of <8 week (*Q*_B_ = 8.779; p = 0.003).The subgroup analysis of the executive function improvement showed the effect size was significantly larger of the weekly training session revealed that ≧3 times per week than that of the weekly training session <3 times per week (*Q*_B_ = 4.132; p = 0.042), and the total number of training sessions ≧24 sessions for executive function is also better than that of <24 sessions (*Q*_B_ = 4.225; p = 0.040) (Table 2).

**Table 2:** Subgroup analysis on overall cognitive function, memory, attention and executive function.

Overall cognitive function (N = 14)				Memory (N = 20)			Attention (N = 20)			Executive function (N = 22)		
	Studies	Hedges’s g	P	Studies	Hedges’s g	P	Studies	Hedges’s g	P	Studies	Hedges’s g	P
**Intervention format**												
Individual	5	0.268	0.285	10	0.411	0.492	10	0.285	0.153	11	0.573	0.124
Group	9	0.522		10	0.326		10	0.160		11	0.284	
**Length of training (mins)**												
<60	7	0.570	0.249	8	0.441	0.462	7	0.314	0.315	7	0.390	0.740
≧60	7	0.310		11	0.336		13	0.194		14	0.460	
undisclosed				1						1		
**Weekly training sessions**												
<3	4	0.395	0.926	10	0.325	0.417	10	0.230	0.895	11	0.259	0.042*
≧3	9	0.420		9	0.432		10	0.216		10	0.647	
undisclosed	1			1						1		
**Total week**												
<8	3	0.582	0.540	8	0.320	0.494	5	0.089	0.003*	9	0.321	0.402
≧8	11	0.391		12	0.400		15	0.288		13	0.486	
**Total session (session)**												
<24	6	0.496	0.553	10	0.280	0.097	14	0.171	0.144	14	0.289	0.040*
≧24	7	0.346		9	0.487		6	0.315		7	0.697	
undisclosed	1			1						1		

### Meta-regression analysis of the characteristics of interventions

Meta-analysis of the effect of the content characteristics of cognitive-based training also indicated that the total number of training weeks (p = 0.048) showed significant effect with attention. The number of weekly training sessions (p = 0.000) and total number of training sessions (p = 0.006) showed significant effect on executive function.

## Discussion

Based on the results of this meta-analysis, we confirmed that cognitive-based training can indeed enhance overall cognitive function and executive function in healthy older people with moderate effect. The cognitive-based training can also enhance other cognitive functional domains, including memory, attention, and visual-spatial ability with small effect.

The results of this study are consistent with previous research on the effect of cognitive-based training for healthy older adults or persons with mild cognitive impairments in overall cognitive function [**15, 30, 31**] and executive function [**17, 32, 33**]. Because the moderate effect on these two indicators were confirmed, the cognitive-based training may therefore relatively effective in enhancing overall cognitive function or task-specific executive function. The results of this study are confirmed that intervention of cognitive-based training had a significant and small effect on memory [**34, 35**], attention [**34, 36**], and visual-spatial ability [**16**]. Cognitive functions associated with crystallized cognition acquired through education and learning, such as verbal knowledge and comprehension, are relatively uninfluenced by aging, while cognitive functions associated with fluid cognition dependent on the brain's processing and response speed are clearly affected by aging. Since these three cognitive function domains may belong to fluid cognition, they tend to deteriorate significantly with increasing age. Many hypotheses have been proposed to explain the effect of aging on fluid cognition in terms of brain mechanisms, but most of these hypotheses fall into two major categories. One view is that aging occurs throughout the brain, and this impedes the real-time integration of earlier and later data when processing information, which then results in slower responses. This is consistent with the empirical finding that certain memory functions tend to be relatively strongly affected by aging. On the other hand, because the memory training interventions among older adults with no cognitive impairment seek to improve memory skills by teaching mnemonic techniques or entail instruction in how to take advantage of environmental supports [**37**], the cognitive-based training may not specify enough to improve it.

Our subgroup findings suggest cognitive-based training showed better effectiveness with weekly training session ≧3 times per week, total training weeks ≧8 weeks, and total training session ≧24 session for healthy older people. Our meta-regression findings also indicated that increases the number of weekly training sessions, total number of training weeks and total number of training sessions can boost attention and executive function; this suggests that increases in the frequency of cognitive-based training can make training more effective[**11, 38**]. These findings are generally consistent with those of previous studies, which have concluded that minimum dose of 15 hours total per targeted brain function, performed over 8 weeks is necessary for real improvement [**39**]. For the training type, cognitive training with a theoretical foundation and specific hypotheses, and is more effective than cognitive stimulation that seek to strengthen mental functioning but have no specific goals.

## Conclusions

The results of this study confirmed that cognitive-based training has a moderate effect on overall cognitive function and executive function, and a small effect on memory, attention, and visual-spatial ability. Subgroup analysis suggest that the intervention frequency with weekly training session ≧3 times per week, total training weeks ≧8 weeks, and total training session ≧24 session is more effectiveness for healthy older people to boost attention and executive function.

Strength of this study are: (1) We applied a strict set of inclusion guidelines; and (2) Performed subgroup analysis of characteristics variables and correlated the difference in the effect size of each study characteristic variable at various levels to explore the effect size. The limitation of this study was that the collected studies had inconsistent research designs. These differences affected effect size and results in the overall analysis, subgroup analysis, and meta-regression.

We recommend that future research pay more attention to psychological, cultural, economic, and other social reasons for participant outcomes, since these outcomes are closely connected with daily lives. Furthermore, the fact that most studies of cognitive-based training had different subsequent tracking periods ensured that the tracking of effectiveness was imprecise. We therefore recommend that, apart from administering immediate post-tests, future research on cognitive-based training should administer a regular tracking test every three months. This would provide future researchers with a better understanding of subsequent effectiveness when performing meta-analyses on this subject.

As for clinical practice, this study suggest that cognitive-based training can promote overall cognitive function, memory, attention, executive function and visual-spatial ability in healthy older people individuals. Consequently, we recommend training sessions at least three times per week, total training weeks of at least eight weeks, and at least 24 total training sessions in the design for the cognitive-based training protocol for the healthy older people to boost attention and executive function. Finally, we hope that this study's findings will provide information about the use of cognitive-based training in healthy older people individuals, and help the healthy older people obtain the greatest possible benefit in terms of health promotion and disease prevention.

## Supporting information

S1 Table PRISMA checklist. (DOC)

S2 Table Studies included in this analysis. (DOCX)
