# Supplementary material for: The effect of cognitive-based training for the healthy older people: A meta-analysis of randomized controlled trials
Source: PLoS One. 2017 May 1;12(5):e0176742. doi: 10.1371/journal.pone.0176742 (PMC5411084; doi:10.1371/journal.pone.0176742)
Supplement: S2 Table — (DOCX) [file pone.0176742.s002.docx]

S2. Table. Studies included in this analysis.

| **NO.** | **Study citation** (author/year) | **Publication** |
| --- | --- | --- |
|  | Ball et al. /2002 | Ball K, Berch DB, Helmers KF, Jobe JB, Leveck MD, Marsiske M, et al. Effects of cognitive training interventions with older adults: a randomized controlled trial. JAMA. 2002;288(18):2271-81. PubMed PMID: 12425704. |
|  | Ballesteros et al. /2015 | Ballesteros S, Mayas J, Prieto A, Toril P, Pita C, de Leon LP, et al. A randomized controlled trial of brain training with non-action video games in older adults: Results of the 3-month follow-up. Front Aging Neurosci. 2015;7:45. PubMed PMID: 25926790. |
|  | Basak et al. /2008 | Basak C, Boot WR, Voss MW, Kramer AF. Can training in a real-time strategy video game attenuate cognitive decline in older adults? Psychol Aging. 2008;23(4):765-77. PubMed PMID: 19140648. |
|  | Blackwood et al. /2016 | Blackwood J, Shubert T, Fogarty K, Chase C. The Impact of a Home-Based Computerized Cognitive Training Intervention on Fall Risk Measure Performance in Community Dwelling Older Adults, a Pilot Study. J Nutr Health Aging. 2016;20(2):138-45. PubMed PMID: 26812509. |
|  | Bozoki et al. /2013 | Bozoki A, Radovanovic M, Winn B, Heeter C, Anthony JC. Effects of a computer-based cognitive exercise program on age-related cognitive decline. Arch Gerontol Geriatr. 2013;57(1):1-7. PubMed PMID: 23542053. |
|  | Cheng et al. /2012 | Cheng Y, Wu W, Feng W, Wang J, Chen Y, Shen Y, et al. The effects of multi-domain versus single-domain cognitive training in non-demented older people: a randomized controlled trial. BMC Med. 2012;10:30. PubMed PMID: 22453114. |
|  | Craik et al. /2007 | Craik FIM, Winocur G, Palmer H, Binns MA, Edwards M, Bridges K, et al. Cognitive rehabilitation in the elderly: Effects on memory. J Int Neuropsychol Soc. 2007;13(1):132-42. PubMed PMID: 17166312. |
|  | Edwards et al. /2015 | Edwards JD, Valdés EG, Peronto C, Castora-Binkley M, Alwerdt J, Andel R, et al. The Efficacy of InSight Cognitive Training to Improve Useful Field of View Performance: A Brief Report. J Gerontol B Psychol Sci Soc Sci.2015;70(3):417-22. PubMed PMID: 24211819. |
|  | Garcia-Campuzano et al. /2014 | Garcia-Campuzano MT, Virues-Ortega J, Smith S, Moussavi Z. Effect of cognitive training targeting associative memory in the elderly: a small randomized trial and a longitudinal evaluation. J Am Geriatr Soc. 2013;61(12):2252-4. PubMed PMID: 24329837. |
|  | Kawashima /2013 | Kawashima R. Mental exercises for cognitive function: clinical evidence. J Prev Med Public Health. 2013;46:S22-S27. PubMed PMID: 23412645. |
|  | Kim et al. /2015 | Kim GH, Jeon S, Im K, Kwon H, Lee BH, Kim GY, et al. Structural brain changes after traditional and robot-assisted multi-domain cognitive training in community-dwelling healthy elderly. PloS one. 2015;10(4):e0123251. PubMed PMID: 25898367. |
|  | Kwok et al. /2013 | Kwok T, Wong A, Chan G, Shiu YY, Lam KC, Young D, et al. Effectiveness of cognitive training for Chinese elderly in Hong Kong. Clin interv aging. 2013;8:213-9. PubMed PMID: 23440076 |
|  | Lee et al. /2013 | Lee TS, Goh SJA, Quek SY, Phillips R, Guan C, Cheung YB, et al. A brain-computer interface based cognitive training system for healthy elderly: a randomized control pilot study for usability and preliminary efficacy. PloS one. 2013;8(11):e79419. PubMed PMID: 24260218. |
|  | Lee et al. /2015 | Lee TS, Quek SY, Goh SJA, Phillips R, Guan C, Cheung YB, et al. A pilot randomized controlled trial using EEG-based brain–computer interface training for a Chinese-speaking group of healthy elderly. Clin Interv Aging. 2015;10:217-27. PubMed PMID: 25624754. |
|  | Legault et al. /2011 | Legault C, Jennings JM, Katula JA, Dagenbach D, Gaussoin SA, Sink KM, et al. Designing clinical trials for assessing the effects of cognitive training and physical activity interventions on cognitive outcomes: the Seniors Health and Activity Research Program Pilot (SHARP-P) study, a randomized controlled trial. BMC Geriatr. 2011;11:27. PubMed PMID: 21615936. |
|  | Linde et al. /2014 | Linde K, Alfermann D. Single versus combined cognitive and physical activity effects on fluid cognitive abilities of healthy older adults: a 4-month randomized controlled trial with follow-up. J Aging Phys Act. 2014;22(3):302-13. PubMed PMID: 23881448. |
|  | Mahncke et al. /2006 | Mahncke HW, Connor BB, Appelman J, Ahsanuddin ON, Hardy JL, Wood RA, et al. Memory enhancement in healthy older adults using a brain plasticity-based training program: a randomized, controlled study. Proc Natl Acad Sci U S A. 2006;103(33):12523-8. PubMed PMID: 16888038. |
|  | Margrett et al. /2006 | Margrett JA, Willis SL. In-home cognitive training with older married couples: individual versus collaborative learning. Neuropsychol Dev Cogn B Aging Neuropsychol Cogn. 2006;13(2):173-95. PubMed PMID: 16807197. |
|  | Millán-Calenti et al. /2015 | Millán-Calenti JC, Lorenzo T, Núñez-Naveira L, Buján A, Rodríguez-Villamil JL, Maseda A. Efficacy of a computerized cognitive training application on cognition and depressive symptomatology in a group of healthy older adults: A randomized controlled trial. Arch Gerontol Geriatr. 2015;61(3):337-43. PubMed PMID: 26321734. |
|  | Mozolic et al. /2011 | Mozolic JL, Long AB, Morgan AR, Rawley-Payne M, Laurienti PJ. A cognitive training intervention improves modality-specific attention in a randomized controlled trial of healthy older adults. Neurobiol Aging. 2011;32(4):655-668. PubMed PMID: 19428142. |
|  | Nouchi et al. /2012 | Nouchi R, Taki Y, Takeuchi H, Hashizume H, Akitsuki Y, Shigemune Y, et al. Brain training game improves executive functions and processing speed in the elderly: A randomized controlled trial. PLoS One, 2012;7(1): e29676. PubMed PMID: 22253758. |
|  | Park et al. /2014 | Park SH, Seo JH, Kim YH, Ko MH. Long-term effects of transcranial direct current stimulation combined with computer-assisted cognitive training in healthy older adults. Neuroreport. 2014;25(2):122-6. PubMed PMID: 24176927. |
|  | Shatil et al. /2013 | Shatil E. Does combined cognitive training and physical activity training enhance cognitive abilities more than either alone? A four-condition randomized controlled trial among healthy older adults. Front Aging Neurosci. 2013;5:8. PubMed PMID: 23531885. |
|  | Shatil et al. /2014 | Shatil E, Mikulecká J, Bellotti F, Bureš V. Novel television-based cognitive training improves working memory and executive function. PLoS One. 2014;9(7):e101472. PubMed PMID: 24992187. |
|  | Smith et al. /2009 | Smith GE, Housen P, Yaffe K, Ruff R, Kennison RF, Mahncke HW, et al. A cognitive training program based on principles of brain plasticity: results from the Improvement in Memory with Plasticity-based Adaptive Cognitive Training (IMPACT) study. J Am Geriatr Soc. 2009;57(4):594-603. PubMed PMID: 19220558. |
|  | Song et al. /2009 | Song MS, Kwon DY, Seo WK, Lim KS, Park MH. The effects of cognitive training on community-dwelling elderly Koreans. J Psychiatr Ment Health Nurs. 2009;16(10):904-909. PubMed PMID: 19930364. |
|  | Stine-Morrow et al. /2008 | Stine-Morrow EA, Parisi JM, Morrow DG, Park DC. The effects of an engaged lifestyle on cognitive vitality: a field experiment. Psychol Aging. 2008;23(4):778-86. PubMed PMID: 19140649. |
|  | Suzuki et al. /2014 | Suzuki H, Kuraoka M, Yasunaga M, Nonaka K, Sakurai R, Takeuchi R, et al. Cognitive intervention through a training program for picture book reading in community-dwelling older adults: a randomized controlled trial. BMC Geriatr. 2014;14:122. PubMed PMID: 25416537. |
|  | Talib et al. /2008 | Talib LL, Yassuda MS, Diniz BS, Forlenza OV, Gattaz WF. Cognitive training increases platelet PLA2 activity in healthy elderly subjects. Prostaglandins Leukot Essent Fatty Acids. 2008;78(4-5):265-9. PubMed PMID: 18467085. |
|  | Wolinsky et al. /2013 | Wolinsky FD, Vander Weg MW, Howren MB, Jones MP, Dotson MM. A randomized controlled trial of cognitive training using a visual speed of processing intervention in middle aged and older adults. PLoS one. 2013;8(5):e61624. PubMed PMID: 23650501. |
|  | Yoon Mi et al. /2013 | Yoon Mi L, Chel J, In Hye B, Joo Soo Y. Effects of Computer-assisted Cognitive Rehabilitation Training on the Cognition and Static Balance of the Elderly. J Phys Ther Sci. 2013;25(11):1475-7. PubMed PMID: 24396214. |
